# Retraction: Neural correlates of handwriting effects in L2 learners

**DOI:** 10.3389/fpsyg.2024.1375201

**Published:** 2024-02-05

**Authors:** Frontiers Editorial Office

The journal retracts the 14th July 2022 article cited above.

Following publication, the authors contacted the Editorial Office to request the retraction of the cited article, stating that the paper contains several serious errors in the experimental design, and in the reporting of both the methodology and data.

The conclusions reported in the article are therefore no longer supported by the analyses/data. An investigation, conducted in accordance with Frontiers policies, confirmed this issue; the article is therefore retracted.

This retraction was approved by the Chief Editors of Frontiers in Psychology and the Chief Executive Editor of Frontiers. The authors agree to this retraction.

